# Thymol screening, phenolic contents, antioxidant and antibacterial activities of Iranian populations of *Trachyspermum ammi* (L.) Sprague (Apiaceae)

**DOI:** 10.1038/s41598-022-19594-7

**Published:** 2022-09-19

**Authors:** Mahdieh Modareskia, Mohammad Fattahi, Mohammad Hossein Mirjalili

**Affiliations:** 1 Department of Horticulture, Faculty of Agriculture, Urmia University, Urmia, Iran; 2 Department of Agriculture, Medicinal Plants and Drugs Research Institute, Shahid Beheshti University, Tehran, 1983969411 Iran

**Keywords:** Biochemistry, Biological techniques, Ecology, Microbiology, Physiology, Plant sciences

## Abstract

The seeds of *Trachyspermum ammi* were gathered at the ripening stage from different regions of Iran and grouped into 14 populations (P1-P14) accordingly. The essential oil (EO) extraction yielded in the 3.16–5% range. EOs were analyzed by gas chromatography-flame ionization detection (GC-FID) and gas chromatography-mass spectrometry (GC–MS) and 11 constituents were identified. Thymol (59.92–96.4%), *p*-cymene (0.55–21.15%), γ-terpinene (0.23–17.78%), and carvacrol (0.41–2.77%) were the major constituents. The highest contents of thymol and carvacrol were found in the Ghayen population (P2). Also, P2 and P8 (Estahban) had the highest value of total phenol (TPC) 43.2 mg gallic acid equivalent (GAE)/g DW, and total flavonoids (TFC) 8.03 mg quercetin equivalent (QE)/g DW, respectively. P1 (Kalat) had the highest total coumarin (TCC) value (0.26 mg coumarin equivalent CE/g DW). Based on EO constituents, principal component analysis (PCA) and cluster analysis classified populations into two chemotypes of thymol/*p*-cymene/γ-terpinene and thymol/carvacrol. The highest positive correlation coefficient was between α-terpinene and limonene (0.96), while the highest negative correlation was between thymol and *p*-cymene (–0.984). The antioxidant activities of extracts and EOs were evaluated by phosphomolybdenum (total antioxidant capacity; TAC), diphenylpicrylhydrazyl (DPPH IC_50_), and ferric ion reducing antioxidant power (FRAP) assays. Also, the antimicrobial activity of EOs was studied against *Escherichia coli* and *Staphylococcus aureus*. P8 with high thymol, EO content (%v/w), TFC, and antibacterial and antioxidant activities is recommended but further studies are needed to confirm the chemotype introduction.

## Introduction

Plants belonging to the Apiaceae family are mostly targeted for large-scale essential oil (EO) production with functional phytochemicals. Among them, ajwain (*Trachyspermum ammi* (L.) Sprague, Synonym: *Carum copticum* (L.) Benth. & Hook.f. ex Hiern) as an annual plant is growing in the east of India, Iran, Pakistan, and Egypt^1,2^. In Iran, the total vegetative herb, fruits (seeds), and roots of the plant are used as a flavoring agent and traditional medicine^3^. Ajwain has been confirmed that has antimicrobial, antioxidant, anticancer, anti-inflammatory, antitermitic, hypotensive, hypolipidemic, antihypertensive, antispasmodic, anti-lithiasis, diuretic, antitussive, nematicidal, antihelminthic, anti-filarial, and insecticidal activities^3–7^. Ajwain essential oil as a potent and natural antimicrobial agent through core–shell electrospun nanofibers structure has been introduced for accelerating infected wound healing^8^. Phytochemicals of ajwain are terpenes, phenolics, alkaloids, flavonoids, glycosides, phytosterols, ascorbic acid, chalcones, coumarins, tannins, steroids, and saponins^9,10^. With regards to a nutraceutical point of view, ajwain seeds are served as valuable constituents in the human diet. *Trachyspermum ammi* with aromatic seeds locally known as Zenian and Jajiq (Jajikh) in Iran. Several studies have reported the chemical composition of ajwain oil with different major constituents including thymol, γ-terpinene, and *p*-cymene^11,12^ carvone, limonene, and dillapiole^13^ and carvacrol and *p*-cymene^14^. However, no chemotype of EO has been reported in this plant, which contains a very high percentage of thymol (> 90%).

The essential oil composition and content in plants depend on several factors such as sexual, seasonal, ontogenetic, genetic variations, ecological, and environmental properties^15,16^. Essential oils (EOs) are used in perfumes, make-up, food preservers, and additives products^17^. Food and pharmaceutical products are mostly enriched with synthetic antioxidants such as butylated hydroxyanisole (BHA), Butylated hydroxytoluene (BHT), and propyl gallate (PG)^18^. However, applying these synthetic antioxidants might lead to serious effects on human health such as toxic and carcinogenic effects^19^. Natural antioxidants in comparison to synthetic ones have been preferred in terms of safety, tolerance, and non-toxicity^20^. Therefore in recent years EOs and extracts have been investigated to replace synthetic antioxidants^21,22^.

Recently phenolic monoterpenes like thymol or carvacrol with strong antioxidant and antibacterial properties are demanded^23^. Thymol, a phenolic monoterpenoid derivative of cymene and isomer of carvacrol is the major constituent in ajwain oil^22^. Thymol and carvacrol (whether chemical or natural) have been considered to be safe by the European Commission and US Food and Drug Administration (FDA) and are classified as flavoring agents; hence, they undergo regulatory requirements as additives for food preservation^24^. There is a little chemical difference between natural and chemical EO, such as changes in optical rotation and deuterium depleted and oxygen-18 (18O) enriched of natural thymol, which can only be determined by special methods like Isotope-ratio mass spectrometry (IRMS)^25^. Research has shown that the antifungal activities of synthetic thymol are lower than natural ones^26^. Natural thymol is mostly obtained from *Thymus vulgaris* L. and *T. ammi*^27^. The other *Thymus* L. species*, Carum copticum*, *Oliveria decumbens* Vent., *Satureja thymbra* L., *Zataria multiflora* Boiss., *Majorana syriaca* (L.) Raf., *Origanum glandulosum* Desf., and *Lippia* L. species were also reported as main sources of thymol with 10.4 to 81.1% in EO^28^.

With increasing thymol use in commercial formulations like mouthwashes, fungicides, pharmaceutical disinfectants, and insecticides, it has become a high-demanded natural antiseptic agent^28^. Also, antioxidant properties can cause the commercialization of natural thymol. Generally, natural disinfectants are obtained from herbs and spices, many of them are used in the human diet to enhance the flavor, color, and aroma of food^29^. In the last years, natural antimicrobials have been interested due to the increased consumer awareness of their quality and safety^30^.

The objectives of the present study are phytochemical screening, and evaluation of antioxidant and antibacterial properties of seeds of *T. ammi* populations in the following items; I) essential oil analysis to find chemotypes with high thymol and their classification; II) evaluation of antioxidant effects of the plant samples EOs and methanolic extracts using 2,2-diphenyl-1-picrylhydrazyl (DPPH), ferric reducing antioxidant power (FRAP), and total antioxidant capacity (TAC) methods; III) evaluation of total phenol, flavonoids and coumarin contents of extract; and finally, IV) evaluation of the antimicrobial activity of EOs against *Escherichia coli* and *Staphylococcus aureus*.

## Results and discussion

### Essential oils yield and composition

Among the 14 seed sample populations collected, the content of EOs among populations ranged from 3.16 to 5% (v/w). The lowest and highest EO content was determined in Ghayen (P2) and Fars (P8) populations, respectively (Table 1). Similarly, the percentage of EO in ajwain samples has been reported from Pakistan 3.5–5.2%^31^, India 2–4%^4,32^, and Iran 2–6%^5,33–35^. EO yield may vary in plants depending on species, quality (chemotype of the plant), condition (fresh or dry), the layout of plant material (e.g., leaf/stem ratio), harvest time, and also extraction method^15,16,36^. The EO yield is an important quality factor to bring medicinal plants to the pharmaceutical, and food industries. Seed EO constituents of the 14 ajwain populations and chromatograms are shown in Table 1 and Fig. S1. In this study, eleven constituents were identified in all 14 populations, and thymol was the major constituent ranging from 59.92 to 96.4 percent (Fig. S2). Other major constituents were *p*-cymene (0.55–21.15%), γ-terpinene (0.23–17.78%), and carvacrol (0.41–2.77%) among populations studied. The highest content of thymol (96.4%) and its structural isomer carvacrol (2.77%) were found in the Ghayen population (P2). Additionally, the lowest thymol content was detected in the Isfahan population (P13) (59.92%). The highest (17.78%) and lowest (0.23%) γ-terpinene content was found in the Isfahan (P13) and Ghayen (P2) populations, respectively. The Birjand population (P3) displayed the highest *p*-cymene content (21.15%) and (P2) showed the lowest content (0.55%).

**Table 1:** The essential oil composition of the fourteen *Trachyspermum ammi* populations.

No	Compounds	Empirical formula	CRI^a^	LRI^b^	P1	P2	P3	P4	P5	P6	P7	P8	P9	P10	P11	P12	P13	P14	Method of identification
1	α-Thujene	C_10_H_16_	926	924	0.02	–	0.13	0.05	–	0.08	0.12	0.01	–	0.08	0.12	0.08	0.22	0.21	RI, MS^c^
2	*α*-Pinene	C_10_H_16_	935	932	0.02	–	0.08	0.1	0.03	0.04	0.1	0.02	–	0.02	0.05	0.04	0.12	0.06	RI, MS
3	β-Pinene	C_10_H_16_	982	974	0.33	0.01	0.76	0.34	0.22	0.27	0.97	0.22	0.29	0.26	0.57	0.61	1.05	0.59	RI, MS
4	β-Myrcene	C_10_H_16_	989	988	0.07	–	0.23	0.18	0.07	0.16	0.27	0.04	0.08	0.11	0.27	0.15	0.39	0.33	RI, MS
5	*α*-Terpinene	C_10_H_16_	1020	1014	0.05	0.01	0.12	0.12	0.04	0.17	0.13	0.04	–	0.08	0.11	0.08	0.23	0.16	RI, MS
6	*p*-Cymene	C_10_H_14_	1031	1020	7.69	0.55	21.15	19.87	8.19	15.66	17.81	4.5	10.08	15.86	20.10	16.17	19.45	20.05	RI, MS, St
7	Limonene	C_10_H_16_	1033	1024	0.07	0.01	0.16	0.12	0.08	0.17	0.15	0.04	0.02	0.07	0.16	0.1	0.24	0.19	RI, MS
8	1,8-cineol	C_10_H_18_O	1036	1026	0.04	–	0.03	0.03	0.05	0.03	0.02	0.03	–	0.01	0.03	0.03	0.03	0.03	RI, MS
9	γ-Terpinene	C_10_H_16_	1062	1054	6.87	0.23	13.02	12.02	6.7	14.55	11.87	3.8	8.93	10.22	13.54	10.64	17.78	14.98	RI, MS
10	Thymol	C_10_H_14_O	1308	1289	83.99	96.4	63.41	66.71	83.38	67.83	68.1	90.57	80.09	72.86	64.42	71.41	59.92	62.96	RI, MS, St
11	Carvacrol	C_10_H_14_O	1311	1298	0.83	2.77	0. 9	0.46	1.23	1.00	0.46	0.72	0.5	0.39	0.61	0.68	0.57	0.41	RI, MS, St
Monoterpene hydrocarbons					15.12	0.81	34.75	32.8	15.33	31.1	31.42	8.67	19.38	26.7	34.92	27.87	39.48	36.57	
Oxygenated monoterpenes					84.88	99.17	64.34	67.2	84.66	68.86	68.58	91.32	80.61	73.26	65.06	72.12	60.52	63.4	
Total identified					99.98	99.98	99.09	100	99.99	99.96	100	99.99	99.99	99.96	99.98	99.99	100	99.97	
Essential oil yield (%)					4.5	3.16	4.83	4.66	3.5	4.33	4.16	5	4.66	4.83	4	3.83	3.5	4	
Thymol yield (%)					3.88	2.97	2.98	3.05	2.85	2.85	2.67	4.25	3.39	3.55	2.59	2.54	1.92	2.54	
γ-Terpinene yield (%)					0.31	0.007	0.61	0.55	0.22	0.61	0.46	0.17	0.37	0.49	0.54	0.37	0.57	0.6	
*P*-Cymene yield (%)					0.35	0.016	0.99	0.91	0.28	0.65	0.69	0.21	0.42	0.77	0.81	0.57	0.62	0.81	

^a^CRI: calculated retention indices determined in the present work relative to *n*-alkanes C6–C24 on DB-5 column.

^b^LRI: literature retention index values (Adams, 2007).

^c^MS: mass spectrum. St; Pure standard co-injection.

**Figure 1:**
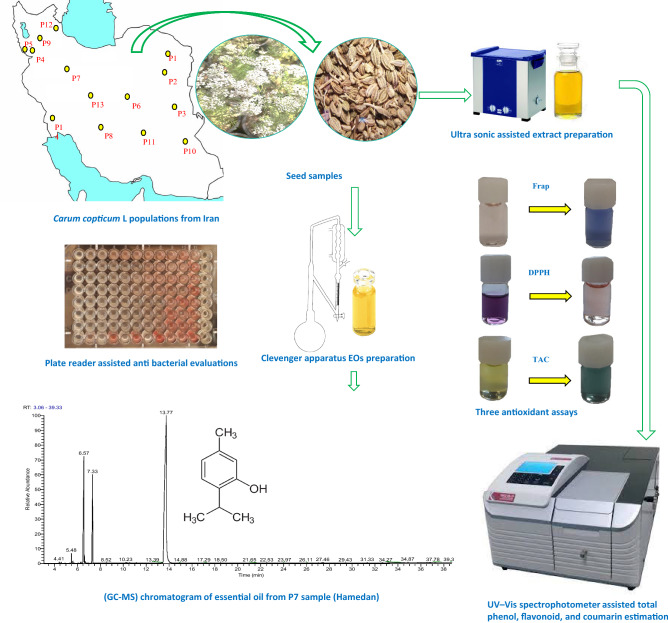
Represent of graphical design of the present research.

The GC–MS spectra obtained from the Hamedan population (P7) are represented in the graphical diagram in Fig. 1. According to our results, the Ghayen population (P2) has the highest levels of thymol and carvacrol and lowest levels of *p*-cymene and γ-terpinene. So, a higher rate of precursors (γ-terpinene and *p*-cymene) to final products (thymol/carvacrol) can be converted in isolated EO^35^. According to the biosynthetic pathway, γ-terpinene precursor converts to thymol and carvacrol during the developmental stages^37^.

**Figure 2:**
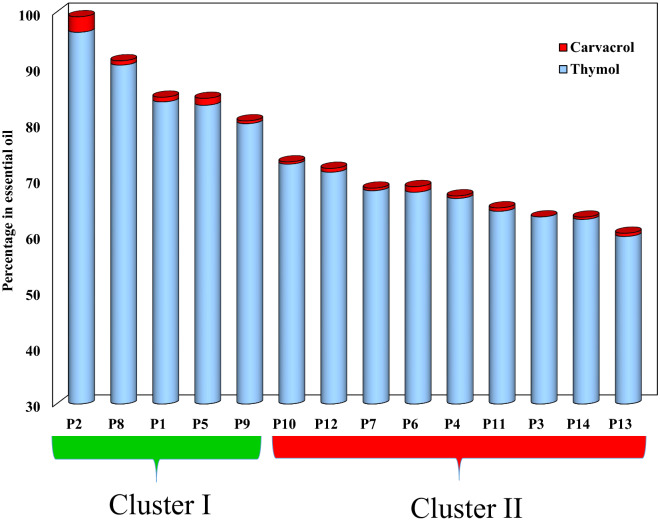
Thymol + carvacrol (%) in EO in studied populations. Chemotype determined with hierarchical cluster analysis (HCA).

In this context, EO compositions of ajwain have been reported from various geographical areas. According to the chemical composition of ajwain oils, major constituents of thymol, γ-terpinene, and *p*-cymene^11,12,33,35^ carvone, limonene, and dillapiole^13^ and carvacrol and *p*-cymene^14^ have been documented. Up to now, the high-thymol content populations from Iran were between 34 to 55%^33^ 48.8 to 61.4^35^, and 65.4^11^. However, no chemotype of the plant EO has been reported with a very high percentage of thymol (> 90%). Thymol and carvacrol percentages of seed EO of 14 populations are shown in Fig. 2. As can be seen in this figure, populations P2 and P8 have the highest thymol content (more than 90% of EO). The presence of a high percentage of thymol in the P8 and P2 can be industrially valuable. Chemotypes are named based on the main constituents in EO within single botanical species^38^. Normally ajwain oils on the market are those rich in thymol and/or carvacrol with strong antibacterial properties and high antioxidant potential. High purity thymol is interested in the market and will not have the subsequent purification costs. Therefore, chemotypes P2 and P8 with a high percentage of thymol 96.4. 90.57% can be significant respectively.

### Estimation of phyto-constituents of extract

**Figure 3:**
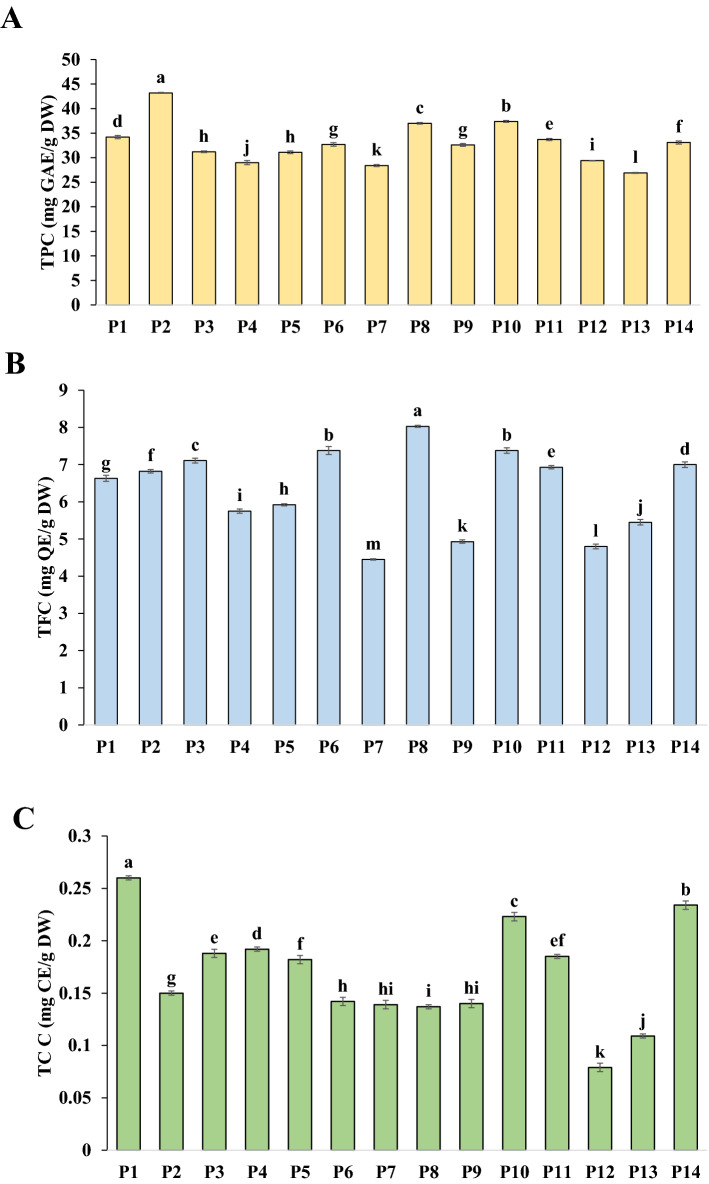
Phyto-constituents analysis of seed samples of 14 studied populations of *Trachyspermum ammi* (**A**); total phenolic content (TPC) as mg Gallic acid/g DW equivalent. (**B**) Total flavonoid content (TFC) quantified based on mg Quercetin/g DW. (**C**) Total coumarin (mg Coumarin E/g DW).

Significant differences were obtained among the population for total phenolic (TPC), total flavonoid (TFC), and total coumarin contents (TCC) (P ≤ 0.01) (Table 2). Natural phenolic compounds are including simple phenolics, phenolic acids, flavonoids, coumarins, tannins, stilbenes, curcuminoids, lignans, quinones, and others^39^. Phenolic compounds and flavonoids are major bioactive components in medicinal plants and thus can comprise an essential part of the human diet^40^. The present study assessed the total phenolic, flavonoid, and coumarin contents of ajwain populations, and the results are presented in Fig. 3A–C. Up to now, no studies have reported total phenol, flavonoid, and coumarin contents of Iranian ajwain populations.

**Table 2:** Analysis of variance for nine phytochemical traits in fourteen populations of *Trachyspermum ammi*.

Source of variation	DF	Mean sum of square								
		TPC (mg GAE/g DW)	TFC (mg QE/g DW)	TCC (mg CE/g DW)	DPPH scavenging IC50 Extract (μg/ml)	DPPH scavenging IC50 E.O (μg/ml)	FRAP Extract (µM Fe^2+^/g DW)	FRAP E.O (mM Fe^2+^/mg EO)	TAC Extract (µM AAE/g DW)	TAC E.O (mM AAE/mg EO)
Population	13	54.3**	3.68**	0.0074**	121.7**	0.233**	0.581**	3.63**	2.198**	17.80**
Error	28	0.017	0.001	0.000005	0.008	0.0001	0.0023	0.015	0.0021	0.0071
C.V. (%)		0.39	0.50	1.29	0.49	0.47	1.69	1.56	1.57	0.70

*TPC* total phenolic content, *TFC* total flavonoid content, *TC* total coumarin, *EO* essential oil.

**Significant at 1% probability level.

### Total phenol content (TPC)

The total phenolic content in the evaluated extracts varied from 26.91 (P13) in the Isfahan population to 43.20 (P2) mg GAE/g DW in the Ghayen population, Results demonstrated that TPC in the populations varied as the following the order P2 > P10 > P8 > P1 > P11 > P14 > P6, P9 > P3, P5 > P4 > P7 > P12 > P13 (Fig. 3A). In the few evaluable sources, the total phenolic content of ajwain seeds extracted with CHCl_3_: MeOH (1: 2) solvent was 69 mg/g DW^41^. In the present study, the highest phenol content (43.2 mg GAE/g DW) was recorded in the P2 population. The difference in TPC with the available report may be due to genetic diversity and differences in extraction methods. According to the presence of apolar thymol in the seed structure, a combination of polar and non-polar solvents to extract compounds may optimize the extraction performance. Various environmental conditions in different places influence the content and metabolic profile of phenolic compounds in plant populations. It seems that high temperature and high UV radiation levels, and differences in genotypes are the reasons why the Isfahan population has a high content of TPC^15,16^.

### Total flavonoid content (TFC)

Analysis of variance showed a significant difference in TFC content at levels P ≤ 0.01. The total flavonoid contents ranged from 4.45 (P7) in the Hamedan population to 8.03 (P8) mg QE/g DW in the Fars population. P6 and P10 with 7.38 mg QE/g DW were also among the high content TFC populations (Fig. 3B). It seems that the reason for the lack of total flavonoids in Hamedan is due genetic differences and the low temperature of this region compared to other regions. Also, the reason for the high level of flavonoids in the Fars population may be due to genetic differences and high temperatures during the growing period. It has been reported that seeds and spurts of ajwain contain 0.58 and 1.15 mg/ g FW of TFC respectively^42^. Also, TFC of methanolic extract of *Anethum graveolens* L. (dill) seeds from the Apiaceae family have been reported to be 5.07 (mg QE /g)^43^. Flavonoid accumulation with many protective roles may be influenced by the combination of genetics (i.e., adaptation to local conditions) and environmental effects (i.e., phenotypic plasticity)^44,45^. Flavonoid accumulation rates among geographically different ajwain populations concerning climate can be correlated positively with temperature and UV-B radiation and negatively with precipitation (Chalker-Scott, 1999; Koski and Ashman, 2015).

### Total coumarin content (TCC)

The TCC content of the *T. ammi* populations examined ranges from 0.079 (P12) to 0.26 (P1) mg coumarin equivalent to dry weight. The highest coumarin content was obtained from the methanolic extract of Kalat (P1) (0.260 mg CE/g DW) and the lowest value of coumarin was recorded for the population of Ardabil (Fig. 3C). Seed coumarin levels in populations can result from genetic and environmental differences. It seems that coumarin accumulation is decreased due to the coolness condition in Ardabil city during the seed maturation stage. Ajwain is a coumarin-rich source of coumarins (umbelliferone, scopoletin, xanthotoxin, bergapten) mostly found in its sprouts^46^. However, no literature source was found to report the amount of total coumarin in ajwain seeds. These compounds have valuable medicinal properties, including edema reduction and possible anticancer activity^47^ Furthermore, they are widely used as a flavoring in foods and pastries. Human exposure to coumarin from the diet has been calculated to be around 0.02 mg/kg/day and its maximum daily intake was estimated to be 0.07 mg/kg BW/day^48^.

### Free radical scavenging effects and antioxidant activity of essential oils and extracts

**Figure 4:**
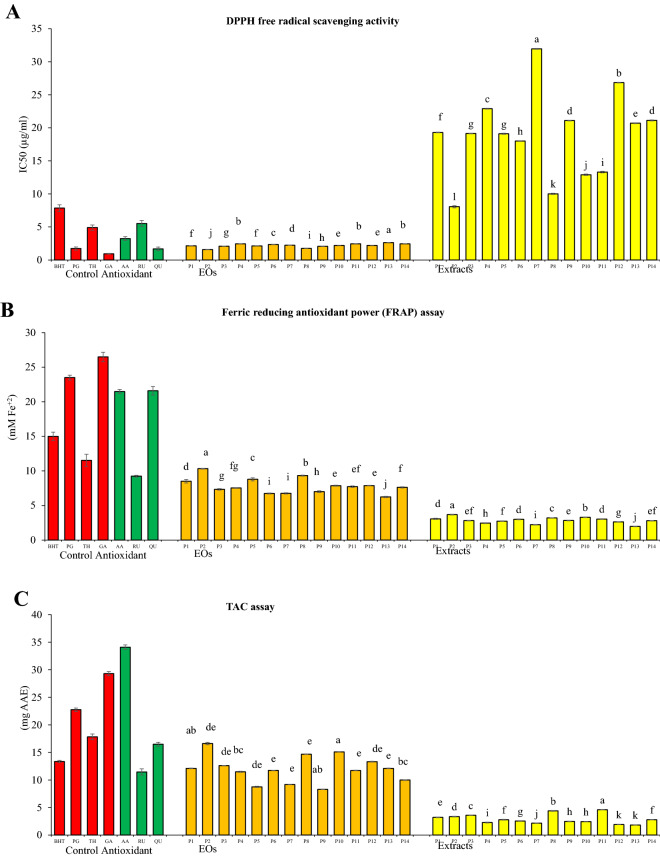
Antioxidant activities of methanolic extracts and essential oils obtained from *Trachyspermum ammi* seed populations and seven antioxidant standards (**A**); Antioxidant activity (DPPH) IC_50_ (µg/ml) (**B**); antioxidant activity (FRAP) quantified by µmol Fe^+2^/g DW (**C**); total antioxidant capacity (TAC) quantified by mg Ascorbic acid equivalent (AAE).

The antioxidant activities of EOs and extracts were assessed using the DPPH, FRAP free-radical scavenging, and total antioxidant capacity (TAC) assays (Fig. 4A–C).

In the DPPH assay, the samples were capable to decrease the DPPH free radical to evaluate their in vitro antioxidant activity. Analysis of variance on DPPH IC50 showed a significant difference in antioxidant activity of EOs and extracts among populations (*P* < 0.01) (Table 2). The value of DPPH (IC 50) in the essential oil varied between (1.57–2.61 µg/ml). The highest rate was related to P13 and the lowest rate was related to P2 samples. Also, the DPPH IC50 in the extract was recorded in the range of 8.06 to 31.95 respectively in P2 and P7. The antioxidant effect of Ajwain EO compared to ascorbic acid has been previously reported. According to this source, an amount of 10 µg of essential oil compared to the same concentration of vitamin C had a DPPH free radical scavenging effect of 76.4 to 97.2%^49^. DPPH radical scavenging activities of the methanolic extract of seeds of *T. ammi* in the range of 30 to 240 µg/ ml have been reported to be 65–80%. While with the same concentrations ascorbic scavenging activities of DPPH were in the range of 90 to 100%^50^. In the comparison of IC50 of EOs and extracts with common antioxidants; ascorbic acid (AA), BHT, PG and rutin (RU), quercetin (QU), gallic acid (GA), and thymol (TH) used in the present study, the following result was obtained from the lowest to the highest. GA < EO (P2) < Qu, PG, EO (P8) < EOs (P9 < P3 < P1 < P10, P12 < P7 < P6 < P4, P11, P14 < P13) < AA < TH < RU < BHT < EXs (P2 < P8 < P10 < P11 < P6 < P3, P5 < P1 < P13 < P9, P14 < P4 < P12 < P7) (Fig. 4A). In the present study, several antioxidants were used to better comparison with essential oils and extracts. BHT, PG, TH, and GA were used as common synthetic antioxidants. The reason for using synthetic thymol was due to comparing it with high thymol content natural studied EO and extracts. Since the plant extract contained flavonoids and phenolic acids, it was tried to use natural phenolic and flavonoid antioxidants for comparison. Ascorbic acid was used in the present study because it is a known and applicable antioxidant. Previously the antioxidant activity of some extracts from the Apiaceae family has been reported. According to these reports, IC_50_ of *Heracleum persicum* Desf., *Prangos ferulacea* (L.) Lindl, *Chaerophyllum macropodum* Boiss., *Oliveria decumbens* extracts were 438, 242, 623, 98.5, and 86.1 (µg/ml), respectively^37,51^. Also based on obtained results, ajwain seed with notable essential oil and extract can be introduced as the new promising antioxidant source from the Apiaceae family.

In the present study, FRAP was used as another method to evaluate antioxidant activity. Based on the results of the analysis of variance, a significant difference was obtained among the population’s EOs and extracts (Table 2). In the EO samples, the highest reducing power was obtained in population 2 (P2), (10.31 mM Fe^+2^) and the lowest was obtained in P13 (6.23 mM Fe^+2^) (Fig. 4B). The reducing power of the extracts was obtained in the range of 1.96 to 3.68 mM Fe^+2^, in which the lowest was related to P13 and the highest to P2. Also, the ferric reducing power of the samples of essential oils, extracts, and standards used in this study were as follows. GA > PG > QU, AA > BHT > TH > EO [P2 > P8) > RU > P5 > P1 > P12 > P14 ≥ P4 ≥ P3 > P9 > P6 > P7 > P13] > EX [P2 > P10 > P8 > P1, P6, P11 > P9 ≥ P14, P3 ≥ P5 > P12 > P4 > P13]. The mechanism by which extracts and EO reduce the [Fe (TPTZ)_2_]^3+^ complex to the ferrous state (Fe^2+^) usually involves the donation of electrons in the form of hydrogen ions and has been related to the in vitro antioxidant activity^52^.

The phosphomolybdenum assay is a quantitative method to evaluate the total antioxidant capacity. Significant differences were obtained in the EO samples as well as the extract samples among the populations (P < 0.01) (Table 2). The values ranged from 8.30 (P9) to 16.61 (P2) (mM AAE/mg EO) in EO samples and from 1.84 (P13) to 4.59 (P11) (µM AAE/g DW) in samples of extracts (Fig. 4C). Results demonstrated that ajwain seeds had notable total antioxidant capacity. The TAC value among antioxidant standards ranged from 11.4 to 34.08 in the following order: AA > GA > PG > TH > QU > BHT > RU. Also, this value ranged from 8.3 to 16.6 among EO samples with the highest value in P2. TCA values in extracts were recorded in the range of 1.83–4.59 with the highest value obtained in P11. Other detailed information is shown in Fig. 4C.

### Antibacterial activity

The antibacterial activity of ajwain EOs was evaluated against two antibiotic resistance bacteria and their ability was compared with Cefixime as a standard. In the present study, we tried to use both gram-positive bacteria and gram-negative bacteria as samples. *Staphylococcus aureus* is a gram-positive pathogenic and antibiotic-resistant bacteria. It is also one of the most common causes of nosocomial infections. Also, *Escherichia coli* is available and inexpensive, and easily cultured in the laboratory. It is one of the most common causes of urinary tract infections. Gram-negative bacteria are also resistant to antibiotics and are an important species in the field of microbiology. One of the main problems in the field of microbiology is the resistance of microbes to antibiotics and so introducing new antibiotics is necessary^53^. The reasons for using Cefixime in the present study are due to its widely used, great therapeutic power, and effectiveness against a wide range of microbes.

In this study, EOs exhibited bacteriostatic activities against *S. aureus* (0.06–64 µg/mL) and *E. coli* (1–64 µg/mL) (Table 3). High thymol content EO (P2) showed high antibacterial activity (MIC = 0.06 µg/mL) against *S. aureus*. Also, the EO from the Isfahan population (P13) showed the lowest antibacterial activity with the highest MIC value (64 µg/mL). In the present study, the mean MIC was not significantly different on gram-negative and positive bacteria, and populations with high thymol had a high antibacterial ability, indicating the antibacterial effects of thymol. Some researchers have evaluated the antimicrobial activity of ajwain oil^14,54,55^. Thymol and carvacrol were found to be more effective in killing bacteria^3–7,9^. The antibacterial properties of natural products, such as essential oils and their components, are widely explored by both industrial and academic fields^56^. The antibacterial activity of the EOs is dependent on the composition and concentration, type, and dose of the target microorganism^57^. The high antibacterial potential of cumin essential oil compared to Ferula essential oil has already been identified due to the high ratio of phenolic monoterpene compounds to other monoterpenes^58^. It seems that the antibacterial effects of *C. copticum* are also mainly due to the presence of phenolic monoterpenes such as thymol, carvacrol, *p*-cymene, and γ-terpinene. Therefore, ajwain EO can be used as a natural agent with antibacterial properties in the food industry and the treatment of infectious diseases, especially antibiotic-resistant strains.

**Table 3:** Minimal Inhibitory Concentrations (MIC) essential oil Iranian 14 populations of Trachyspermum ammi against Escherichia coli and Staphylococcus aureus.

Populations code	MIC^d^ EO^e^ µg/ml	
	*Escherichia coli* PTCC (Gram-negative)	*Staphylococcus aureus* ATCC: 1431 (Gram-positive)
P1	8	2
P2	4	0.06
P3	16	˃ 64
P4	32	16
P5	1	4
P6	32	16
P7	16	32
P8	4	2
P9	1	8
P10	32	8
P11	32	32
P12	8	32
P13	64	> 64
P14	64	4
Cefixime	22.42	20.29

### Hierarchical cluster analysis (HCA) of essential oil constituents

**Figure 5:**
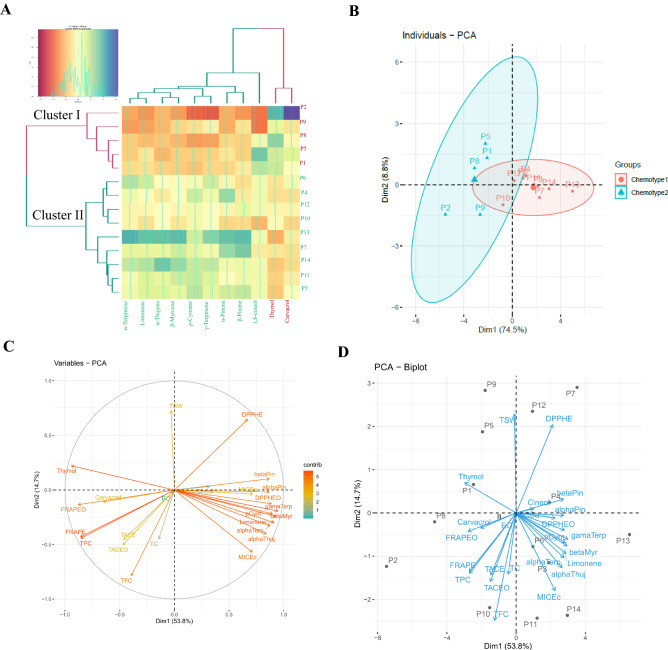
(**A**) Heat-map diagram of two-way hierarchical cluster analysis (HCA) of fourteen *Trachyspermum ammi* populations based on 11 essential oil constituents quantified by GC and GC–MS. Blue color with a great positive share and red color with a great negative share affects cluster formation. (**B**) Principal component analysis (PCA) based on EO constituents. (**C**) PCA is based on all studied traits. (**D**) PCA is based on all studied traits according to populations.

HCA was performed by using the 11 identified compounds and 14 populations (Fig. 5A). All used populations were divided into two clusters; Cluster I included P4, P6, P7, P10, P11, P12, P13, and P14 and cluster II consist of P1, P2, P5, P8, and P9 samples. In cluster I the major constituents were thymol (59.92–72.86), *p*-cymene (15.66–21.15), and γ-terpinene (10.22–17.78). In the second cluster thymol (80.09–96.4) and carvacrol (0.5–2.77) were the major constituents. Cluster analysis can classify studied populations into several groups, according to the chemical composition by ‘magnifying’ their similarities^59^. Forasmuch as, plant sources from environmentally different origins led to the emergence of new chemotypes to baring domestication and cultivation to obtain uniform chemical plants along with appropriate agricultural features^60^.

### Principal component analysis (PCA)

**Figure 6:**
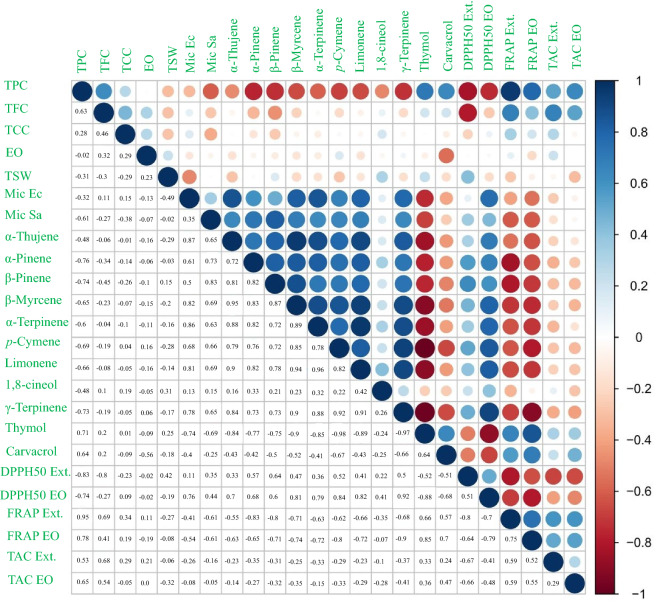
Correlation between 24 traits on the studied *Trachyspermum ammi* populations: TPC: Total phenolic content, TFC: Total flavonoid content, TCC: Total coumarin, EO: Essential Oil yield, TSW: One thousand seed weight (g), MIC: minimum inhibitory concentration, Ec: *E. coli*, MIC: minimum inhibitory concentration, Sa: *S. aureus*, DPPH Ext.: DPPH assay Extract is expressed as IC50 index, DPPH EO: DPPH assay EO is expressed as IC50 index, FRAP Ext.: FRAP assay Extract, FRAP EO: FRAP assay Essential oil, TAC Ext: The total antioxidant capacity Extract, TAC EO: The total antioxidant capacity Essential oil.

Principal component analysis (PCA) is one of the multivariate statistical techniques used to explain differentiation between populations and to obtain more information on the variables that mainly influence the population's similarities and differences^61^. The PCA was performed to identify the most significant variables in the data set (Fig. 5B). The same data set (14 population × 11 components) was used in this section. The PCA showed two components with explain 83.3% of the total variance. The first principal component (PC1) had the most portion of variance (74.5%) which was given by compounds such as γ-Terpinene, α-pinene, α-Thujene, *p*-cymene, and limonene. The second component (PC2), explaining 8.8% of the total variance, consisted of compounds thymol, carvacrol, and 1, 8-cineol (Fig. 6). The results of PCA agreed with those of the cluster analysis the populations similarly were divided into two distinct groups including high thymol/carvacrol and high thymol/p-cymene/γ-terpinene groups (Fig. 5B). Heat map analyses were drowned to determine how constituents effect on clustering. Based on heat map analysis samples were well-classified.

Also, in the analysis of the principal factors (PCA) between all the evaluated traits in the populations, the first principal factor (PC1) showed 53.8% and the second principal factor (PC2) 14.7% of the variance. This analysis determined the principal component, correlation of traits, and their relationship with populations. Accordingly, traits with positive arrows show a positive correlation and two traits with non-directional arrows show a negative correlation. Accordingly, thymol and carvacrol have a high correlation with antioxidant properties and this property is correlated with populations of chemotype 1 (P1, P2, P5, P8, P9). Other relationships and details correlations are shown in Fig. 5C, D.

### Correlation

Simple correlation estimated the relationship between variables. Simple correlations between 24 studied traits in the present study are shown in Fig. 6. Thymol as the major constituent of EOs showed a high positive correlation with TPC (0.71), carvacrol (0.64), FRAP EO (0.85), and FRAP ext. (0.66). Thymol also had a significant negative correlation with Mic EO (-0.74), Mic Sa (-0.69), α-Thujene (-0.84), *α*-Pinene (-0.77), β-Pinene (-0.75), β-Myrcene (-0.9), α-Terpinene (-0.85), *p*-Cymene (-0.98), Limonene (-0.89), γ-Terpinene (-0.97). TPC had a positive correlation with TFC, thymol, carvacrol, FRAP Ext., TAC Ext., and a significant negative correlation with DPPH Ext. The antioxidant methods in extracts DPPH50 vs FRAP (-0.8), DPPH50 vs TAC (-0.67) and FRAP vs TAC (0.59) were highly correlated. Similarly, in estimating the antioxidant activity of essential oil DPPH50 vs FRAP (-0.79), DPPH50 vs TAC (-0.48), and FRAP vs TAC Ext (0.55) were highly correlated. Also, the high correlation of all antioxidant methods with thymol can explain its positive effect on the antioxidant activity of the extracts and EOs. The correlations found between each of the traits can be very important in breeding programs.

## Conclusion

Ajwain (*T. ammi*) is one of the aromatic seed spices and a rich source of specialized metabolites such as thymol. Considering that the plant is one of the main and natural sources of thymol. As natural native Iranian populations of the plant can be the primary sources for breeding and domestication of valuable thymol chemotypes, therefore the present study was conducted to determine high thymol content chemotypes. Based on the results, P2 and P8 populations with a thymol content above 90% are introduced for this aim. Fars population (P8) with high thymol, EO percent, and total flavonoid also high antibacterial and antioxidant activity is recommended for nutraceutical and pharmacological uses. Fourteen populations were classified into two chemotypes of thymol/p-cymene/γ-terpinene type and thymol/carvacrol type. A comparison of the antioxidant effects of EOs with seven synthetic and natural antioxidants used in the present study showed that the plant EOs have stronger antioxidants than these antioxidants. Therefore, EOs of the plant can be used in various industries after supplementary studies. Also, plant extracts are introduced as an antioxidant source for industrial use due to the presence of phenolic and flavonoid compounds. The antibacterial properties of EOs against two bacteria Gram-positive (*S. aureus* ATCC: 1431) and Gram-negative (*E. coli* PTCC: 1399) and their correlation with thymol indicate the importance of high thymol selected chemotypes.

## Methods

### Collection of plant materials

The studied plant materials (seeds of 14T*. ammi* populations) were collected from different regions of Iran. Ecological information, local names and usage, and the weight of one thousand seeds of populations are presented in Table 4. The plant was identified at the herbarium of Medicinal Plants and Drugs Research Institute (MPH) of Shahid Beheshti University, Tehran, Iran, and a voucher specimen (no. MPH-2938) were deposited at MPH (Fig. S3). Since the plant is not an endangered plant, the collection of samples with the permission of the university was done only for academic study by observing the necessary guidelines for collecting plants (IUCN Policy Statement on Research Involving Species at Risk of Extinction and the Convention on the Trade in Endangered Species of Wild Fauna and Flora). Also, except for a few limited herbarium specimens, only a limited percentage of seeds were collected from each population. Figure 1 represented the graphical design of the present research.

**Table 4:** Geographical regions and local name, usage, thousand seed weight of Trachyspermum ammi populations of Iran.

Code	Collection location	Altitude (m)	Latitude (N)	Longitude (E)	Usages in folk medicine	Local name	TSW (g)
P1	Kalat, Razavi Khorasan, E	1150	36°22ʹ25ʺ	59°10ʹ20ʺ	Cooking bread, relieves toothache	Sperkay	1.122
P2	Ghayen, South Khorasan, E	1869	33°43ʹ00ʺ	59°15ʹ00ʺ	Eaten with honey, and vinegar useful for removal of kidney stone	Ajqu	0.642
P3	Birjand, South Khorasan, E	1316	32°46ʹ50ʺ	58°53ʹ39ʺ	Powder with honey for relieves pains, decoction for indigestion	Zinyan	0.841
P4	Mahabad, west Azerbaijan, NW	1320	36°46ʹ03ʺ	45°43ʹ07ʺ	Decoction as anti-helminth and anti-flatulent	Razianakevy	0.715
P5	Sardasht, West Azerbaijan, NW	1435	36°09ʹ17ʺ	45°28ʹ48ʺ	Decoction for removal hiccup, carminative	Razianatouna	0.945
P6	Yazd, Yazd, Center	869	32°11ʹ24ʺ	54°17ʹ24ʺ	Seeds are used as a tea for poison purify	Zenyan, Khord daneh	0.940
P7	Hamedan, Hamedan, W	1791	34°48ʹ23ʺ	48°30ʹ58ʺ	Seeds are eaten with honey, useful for strengthening the liver	Nankhah	1.154
P8	Estahban, Fars, SW	1830	29°06ʹ15ʺ	53°02ʹ45ʺ	Seeds are used in pickles, decoction menstrual regulation	Zenyan	1.122
P9	Maragheh, East Azerbaijan, NW	1485	37°30ʹ35ʺ	46°12ʹ30ʺ	Fines herbs, seed powder on omelet	Jajykh	0.957
P10	Zabol, Sistan and Baluchestan, E	475	31°01ʹ30ʺ	61°25ʹ45ʺ	It uses with Nabat as a tea combination (Rock candy or crystallized sugar) for gastric diseases. Root and seed decoction is used for appetizing and fattening, anti-nausea	Ajowan, Chah ghanbar	0.615
P11	Rafsanjan, Kerman, SE	1529	30°26ʹ30ʺ	56°25ʹ25ʺ	Green vegetable for salad; fruits are sprinkled over bread with black caraway, safflower, and nigella	Kesrk	0.664
P12	Ardebil, Ardebil, NW	1347	38°14ʹ49ʺ	48°17ʹ58ʺ	Leaves as an edible vegetable, cook potage, Plant and decoction for alimentary and respiratory ailments	Jajykh	0.978
P13	Isfahan, Isfahan, Center	1575	32°39ʹ05ʺ	51°40ʹ45ʺ	Decoction of its seeds as an expectorant, belly pains	Zinyan	0.831
P14	Izeh, Khuzestan, S	1070	31°40ʹ27ʺ	50°26ʹ57ʺ	Flavoring fish, meat, and vegetable, it is used with yogurt for foods digestion	Kamun al muluki	0.678

*TSW* one thousand seed weight (g), *E* east; *NW* north-west, *SW* south-west, *SE* southeast, *S* south.

### Extraction of the essential oils (EOs)

To extract EOs, 50 g of each seed source was extracted by a Clevenger-type apparatus for three hours. The EO was then collected in a glass container and kept at 4 °C in the dark condition until analyzed and characterized.

### GC–MS and GC analysis

The obtained EOs was analyzed by gas chromatography-flame ionization detector (GC-FID) and GC-mass spectrometry (GC–MS). The analysis was carried out using a DB-5 fused silica capillary column (length 30 m; inner diameter 0.25 mm; film thickness 0.25 μm). The injector and detector temperatures were kept at 250 and 280 °C, respectively. Helium was used as the carrier gas at a flow rate of 1.1 mL/min; the oven temperature was programmed from 60 to 250 at 5 °C/min and held for 40 min. The injection volume was 1.0 µL using a 1:10 split ratio. GC–MS analysis was carried out with a Thermoquest–Finnigan gas chromatograph equipped with DB-5 fused silica capillary column (length 60 m; inner diameter 0.25 mm; film thickness 0.25 μm) coupled with a TRACE mass spectrometer (Manchester, UK). Helium was used as the carrier gas with a flow rate of 1.1 mL/min. The MS fragmentation was performed by electronic impact (EI) at 70 eV with a scan time of 0.4 s and a mass range was 40–460 amu. The ion source and interface temperatures were 200° and 250 °C, respectively. The oven temperature was the same as above for the GC. The compounds were identified by comparison of their mass spectra with those of the internal reference mass spectra library (Adams and Wiley 7.0) and confirmed by comparison of their retention indices with authentic compounds or with those reported in the literature^62,63^. Retention indices were calculated using the retention times of *n*-alkanes (C_6_–C_24_).

### Extracts preparation

500 mg of seeds were powdered and transferred to test tubes. Twenty mL of methanol (80%) was added to each sample and stirred slightly. Tubes were sonicated for 30 min (Elmasonic EASY 120 H, Germany). The samples were filtered through Whatman filter paper no. 1 and preserved in dark condition at 4 ˚C before assays.

### Total phenolic content (TPC)

The TPC was estimated by the colorimetric Folin-Ciocalteu method^64^. 200 ml of the extracts were mixed with 1200 µL of Folin-Ciocalteu reagent (10%), 180µL of H_2_O, and 960µL sodium carbonate 7%; then this mixture was shaken and incubated at room temperature for 30 min in the darkness. The samples resulting blue color and their absorbance were determined at 765 nm using UV–Vis spectrophotometer (Dynamica HALO DB-20, UK). The TPC was expressed as mg GAE/g dry weight.

### Determination of total flavonoid content (TFC)

The TFC was determined using aluminum chloride colorimetric assay^65^. The extraction solution was combined with 150 μL of 5% sodium nitrite solution after 5 min remaining, 300 μL of aluminum chloride solution (10% w/v), and 1 mL of NaOH (1 M) were added. After incubating the samples for 10 min, the mixture turned pink and the absorbance was recorded at 380 nm by spectrophotometer (Dynamica HALO DB-20, UK). The TFC was calculated as mg of quercetin equivalent (QE) per g of dry weight (DW).

### Determination of total coumarin content (TCC)

The TCC was evaluated based on the Borntrager reaction^66^. 500 μL of the extracts were transferred to a test tube. Afterward, 2 ml of distilled water and 500 μL of lead acetate solution (5%, w/v) were added. The sample is shaken and then 7 ml of distilled water is added. Then 2 mL of prepared mixtures were transferred to a new test tube and add 8 mL of HCl solution (0.1 M, v/v). The samples remain at room temperature for 30 min and then were read at 320 nm by spectrophotometer and the TCC is expressed as milligrams of coumarin equivalents (mg CE/g DW).

### Antioxidant activity assays

The antioxidant activity of the EOs and extracts of seeds was determined by using three assays of DPPH, FRAP, and TAC. The results were compared with standards including BHT (butylated hydroxyl toluene) and PG (Propyl gallate), thymol, gallic acid, ascorbic acid, rutin, and quercetin.

### DPPH radical scavenging assay

The 2, 2-diphenyl-1-picrylhydrazyl (DPPH) free radical scavenging assay was performed by the method with some modification^67^. DPPH scavenging activity of EOs, extracts and control antioxidants were measured. For this means DPPH (100 µM) was dissolved in methanol to prepare the fresh stock solution. The DPPH solution (2 ml) was added to the test compounds and was shaken and incubated in darkness for 30 min at room temperature. The absorbance was monitored at 517 nm against a blank using a UV–Vis spectrophotometer. The inhibition percentage of the DPPH free radical (I %) was calculated as follows:

$${\text{I}}\% \, = \,[({\text{Abs}}_{{{\text{control}}}} {-}{\text{Abs}}_{{{\text{sample}}}} )/{\text{Abs}}_{{{\text{control}}}} ]\, \times \,{1}00$$

Abs_control_, absorbance of DPPH radical; Abs _sample_ is the absorbance of the (DPPH radical + test samples).

I% was plotted against sample concentrations to obtain the IC_50_ index, which was defined as the concentration of antioxidant required to decrease the initial DPPH concentration by 50% DPPH (Brand-Williams et al., 1995).

### Ferric reducing antioxidant power (FRAP) assay

The FRAP assay was determined using the reducing power technique^68^. The samples with antioxidant properties will reduce the ferric ion (Fe^3+^) to the ferrous ion (Fe^2+^) in an acidic medium (pH = 3.6), to form an intense blue complex (Fe^2+^/TPTZ). The FRAP reagent was prepared by mixing acetate buffer (300 mM, pH 3.6), a solution of 10 mM TPTZ (Tripyridyl-s-triazine) in 40 mM HCl, and 20 mM FeCl_3_ at a ratio of 10:1:1 (v/v/v). The FRAP reagent (2 mL) was added to test tubes and mixed thoroughly and were incubated at 37 °C in the dark for 30 min. The absorbance of the samples was taken at 593 nm in comparison to a blank. The standard curve was prepared using different concentrations of ferrous sulfate. The results were expressed in µM Fe^2+^/g DW.

### Phosphomolybdenum assay

TAC was evaluated using phosphomolybdenum assay^69^. The method is based on the reduction of Mo (VI) to Mo (V) with the subsequent formation of a phosphate–Mo^5+^ complex. 1 ml of 0.6 M sulfuric acid, 28 mM sodium phosphate, and 4 mM ammonium molybdate was added to 20 ml of distilled water and made up the volume to 50 ml adding distilled water. 50 µL of (EOs, extracts, and standards) were added to each test tube individually containing 1 ml of Molybdate reagent solution. These capped tubes were kept incubated at 95 ˚C for 90 min. The tubes were cooled at room temperature and then their absorbance was measured at 695 nm using a UV–visible spectrophotometer against blank. The antioxidant capacity was expressed as equivalents of ascorbic acid (mg AAE/g extract).

### Antibacterial activity

The antibacterial potential of EOs was tested against two bacteria Gram-positive (*Staphylococcus aureus* ATCC: 1431) and Gram-negative (*Escherichia coli* PTCC: 1399). Minimum inhibitory concentration (MIC) was conducted according to the standard broth microdilution technique following the guideline of the Clinical Laboratory Standard Institute (CLSI)^70^ in 96 well microplates. Microdilution series of EOs were prepared in Mueller–Hinton Broth (from 0.128–06 µg/ml with a final volume of 100 µl). A suspension of fresh culture medium (18–20 h) was prepared in normal saline and turbidity was adjusted to a 0.5 McFarland tube. The suspension was diluted 1:100 with Mueller–Hinton Broth and then 100 μl of it was added to an individual well. Thus assay is performed by applying a bacterial inoculum of 0.5–1 × 10^5^ CFU/ml to each well. With the addition of bacterial suspension, the final concentration of the test substance in each well was halved. After incubation at 37 °C for 24 h, the wells were investigated for turbidity and MIC was determined and recorded in µg/ml. Samples of EOs assessed in a concentration range of 0.03 to 64 µg/ml. Because of turbidity in some samples, the five μl (4 mg/ml) of 0.2 µm filter-assisted sterilized resazurin solution was used to distinguish how wells grow. Culture medium with bacteria suspension as positive control and culture medium as negative control were used. MIC was determined as soon as the color of the positive control well changed. The experiment was performed twice with three replications and cefixime was evaluated as a standard.

### Statistical analysis

We confirmed that all methods were performed in accordance with the relevant guidelines and regulations. All experiments have been carried out in triplicate and are expressed as the mean ± standard deviation (SD). Analysis of variance (ANOVA) was applied as a statistical analysis of phytochemical data. Data analyses were carried out using SAS Version 9.4 statistical software. The comparison of the means was performed by SNK test at a 1% level. Principle component analysis (PCA), cluster analysis, Heat-map, and correlation analysis were obtained using RStudio (version 1.2.5019) URL http://www.rstudio.com/.

## Supplementary Information

Supplementary Information.

## Data Availability

All data generated or analyzed during this study are included in this published article [and its supplementary information files].
