# Supplementary material for: Thymol screening, phenolic contents, antioxidant and antibacterial activities of Iranian populations of Trachyspermum ammi (L.) Sprague (Apiaceae)
Source: Sci Rep. 2022 Sep 19;12:15645. doi: 10.1038/s41598-022-19594-7 (PMC9485261; doi:10.1038/s41598-022-19594-7)
Supplement: Supplementary file 1 — Supplementary Information. [file 41598_2022_19594_MOESM1_ESM.docx]

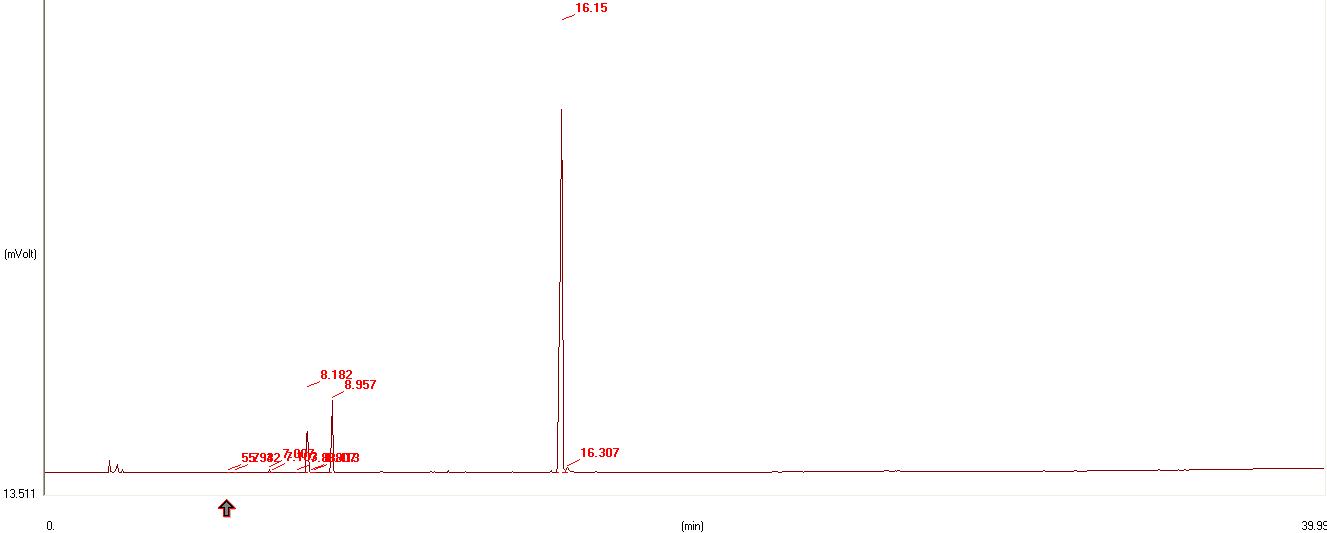

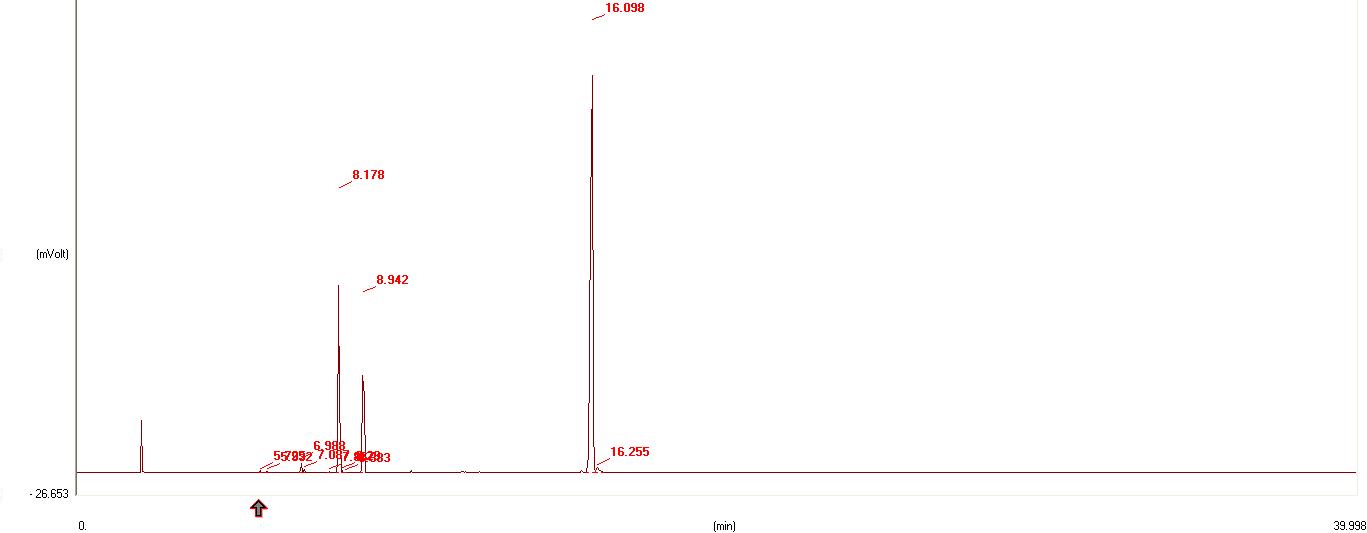

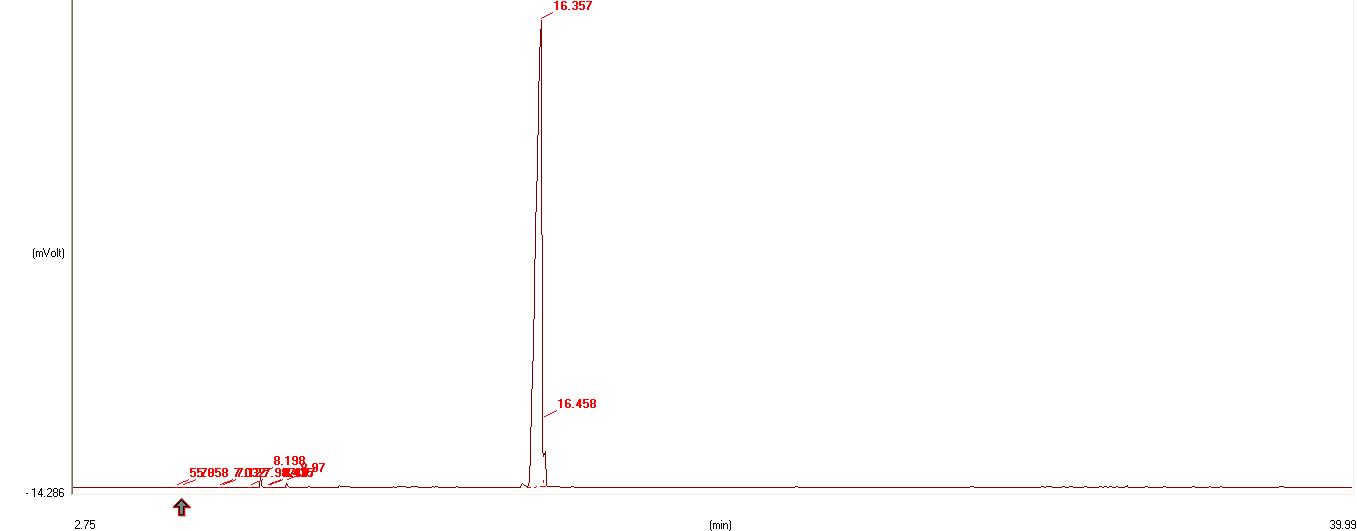


Fig. S1. GC chromatograms of 14 population of *Trachyspermum ammi* collected from different sites of Iran. A-N corresponds to P1- P14.


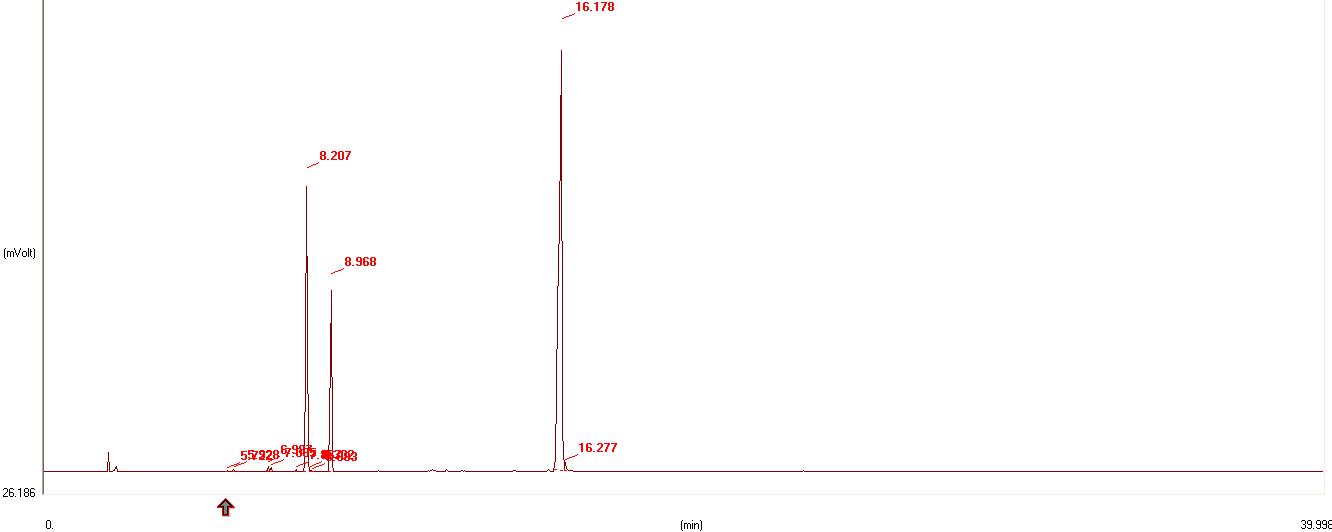

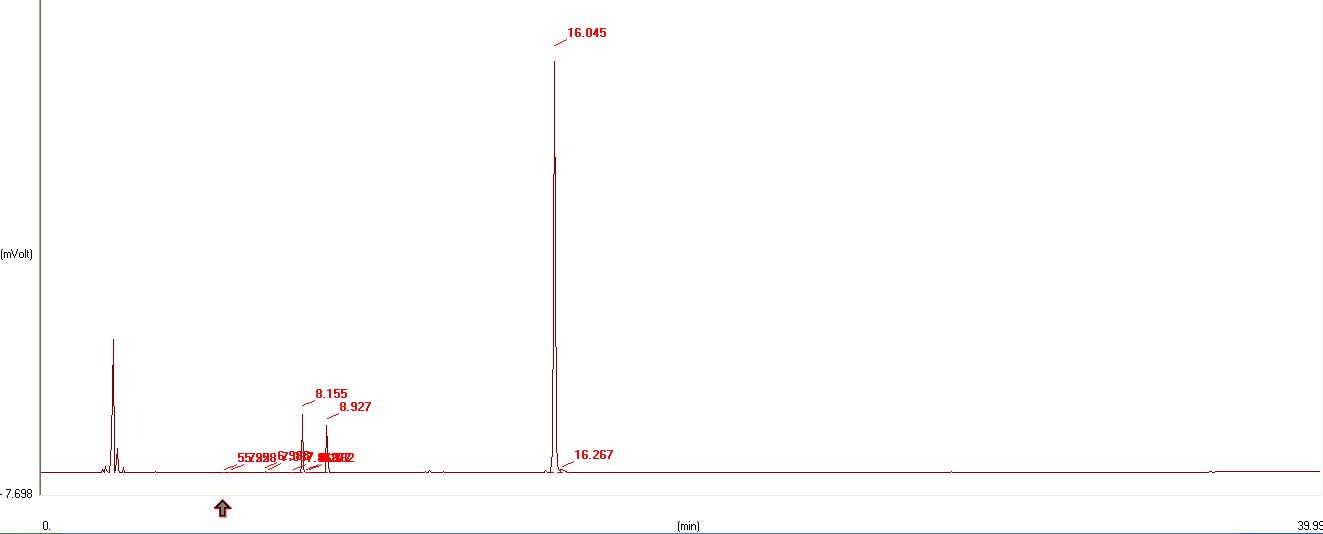

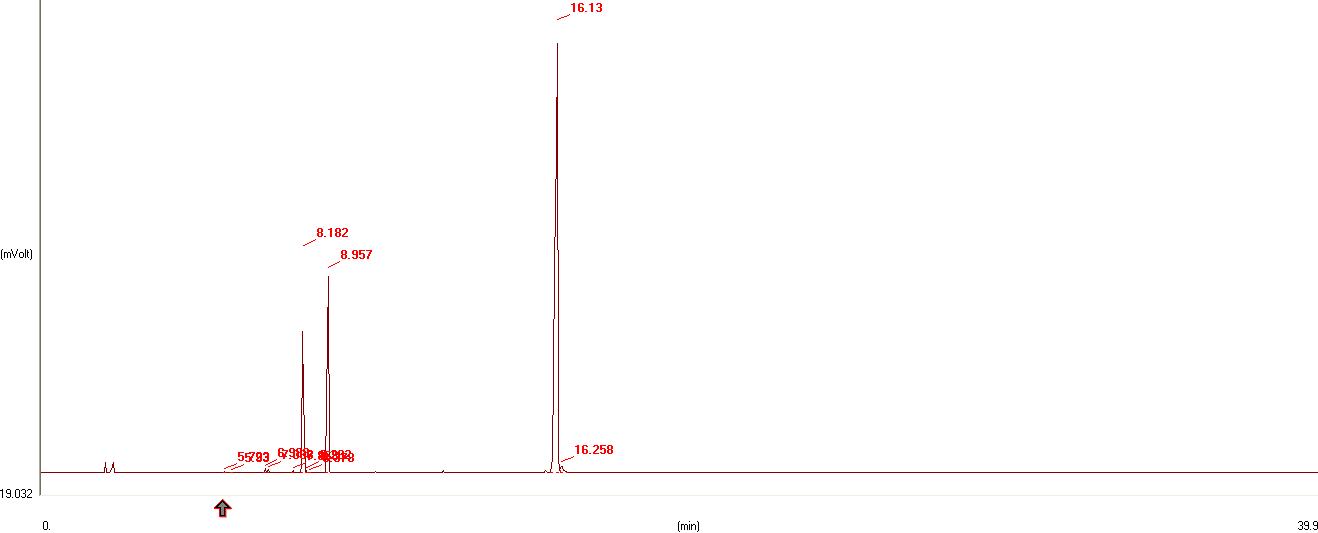


**Fig. S1.** Continued


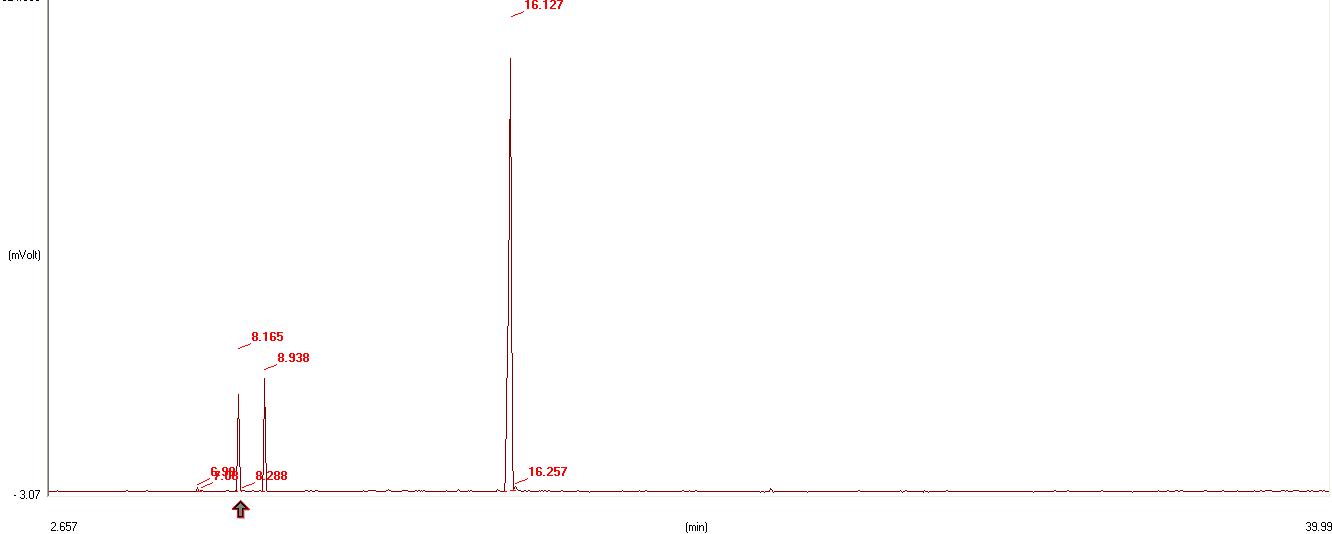

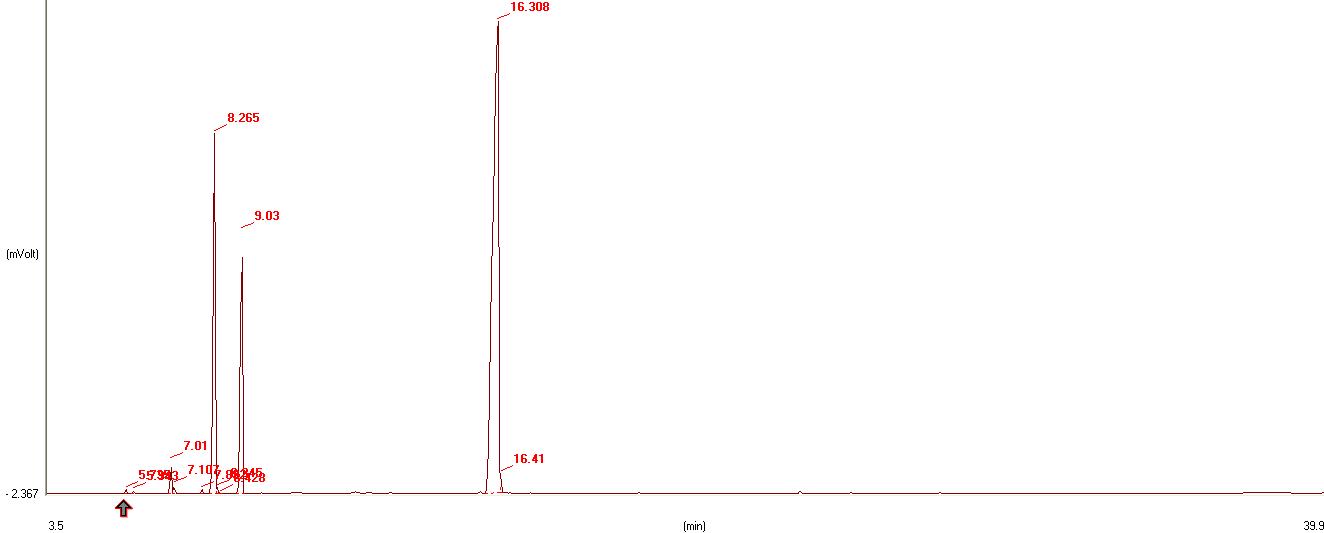

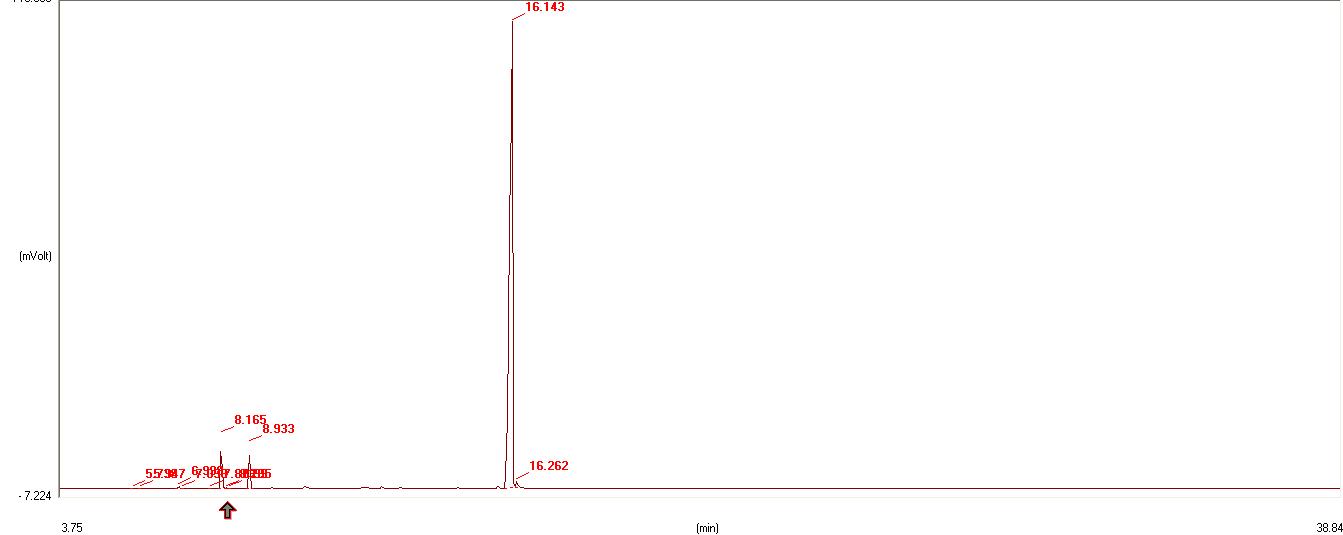


**Fig. S1.** Continued


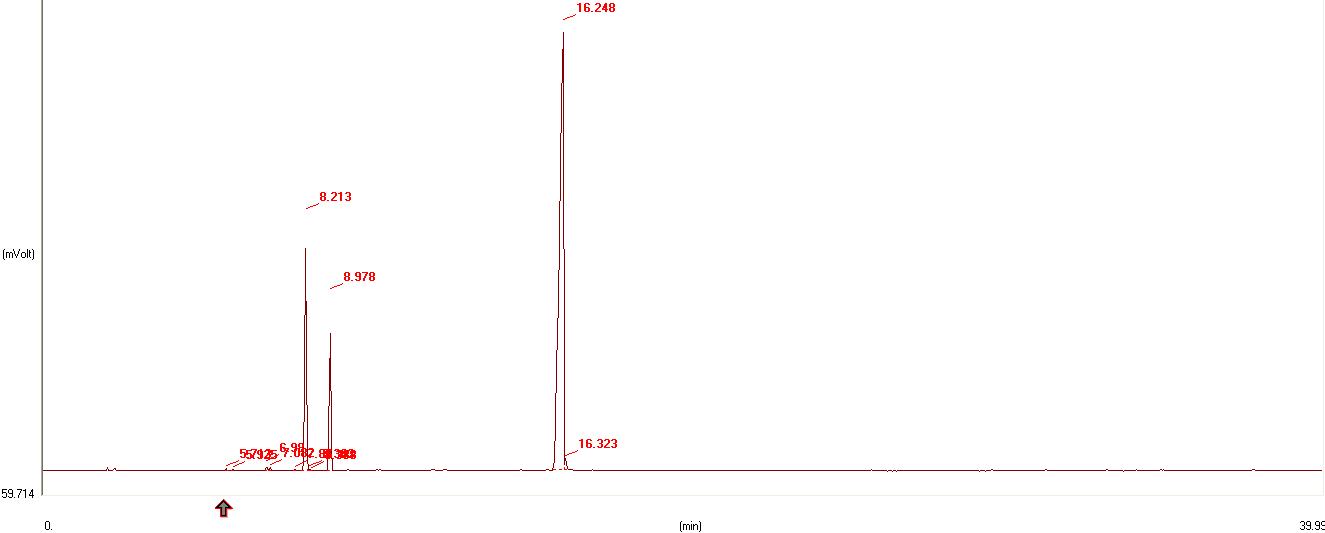

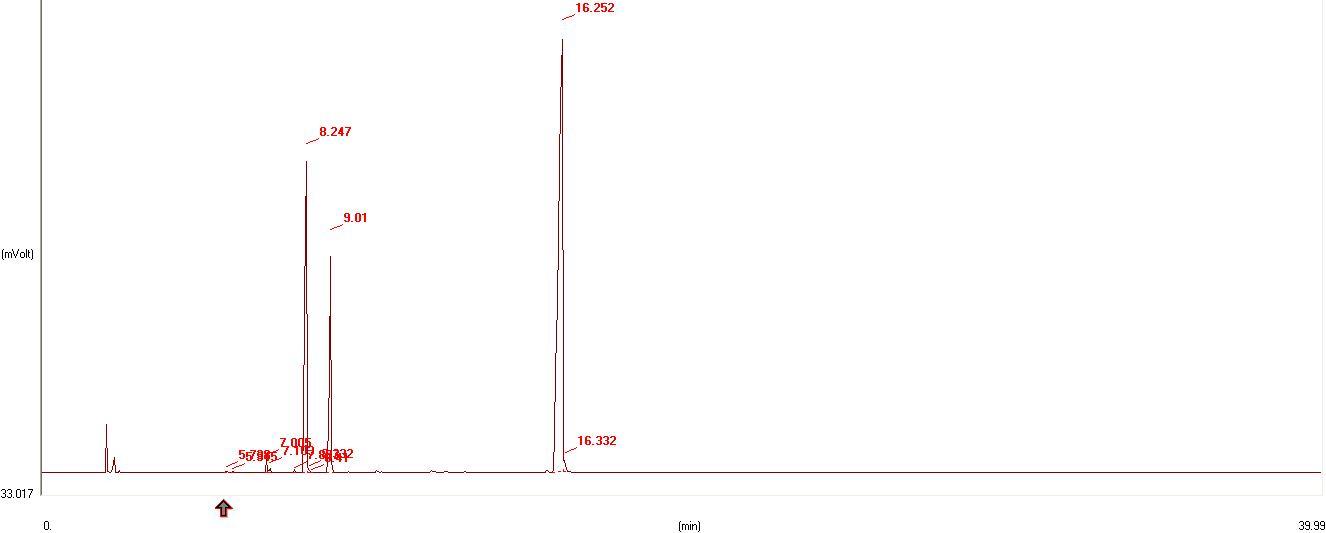

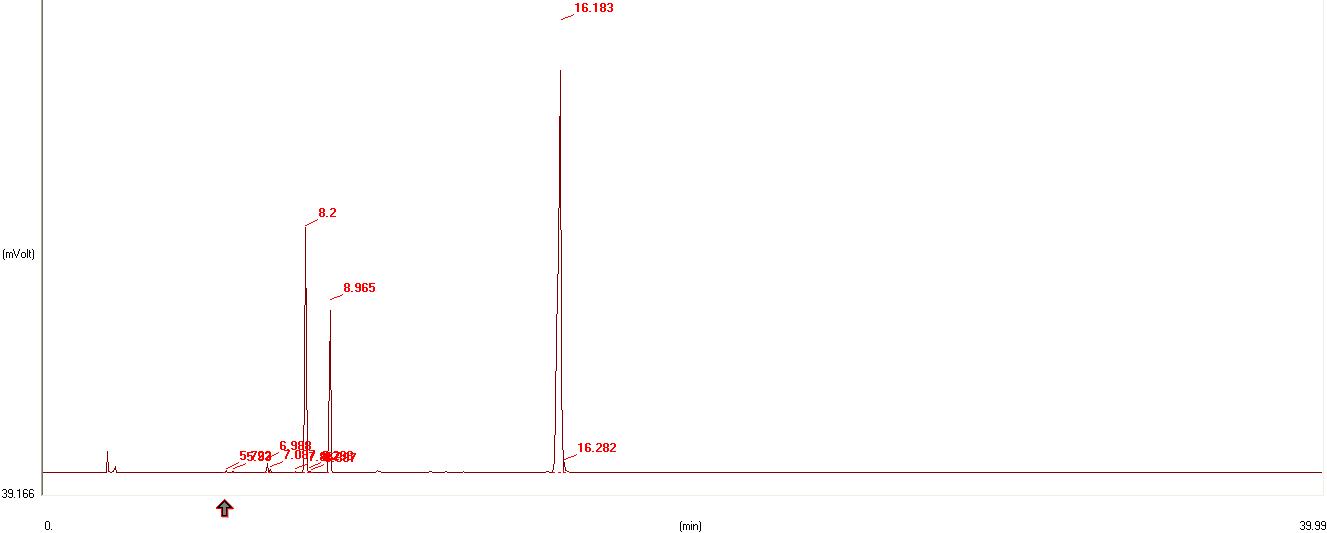


**Fig. S1.** Continued


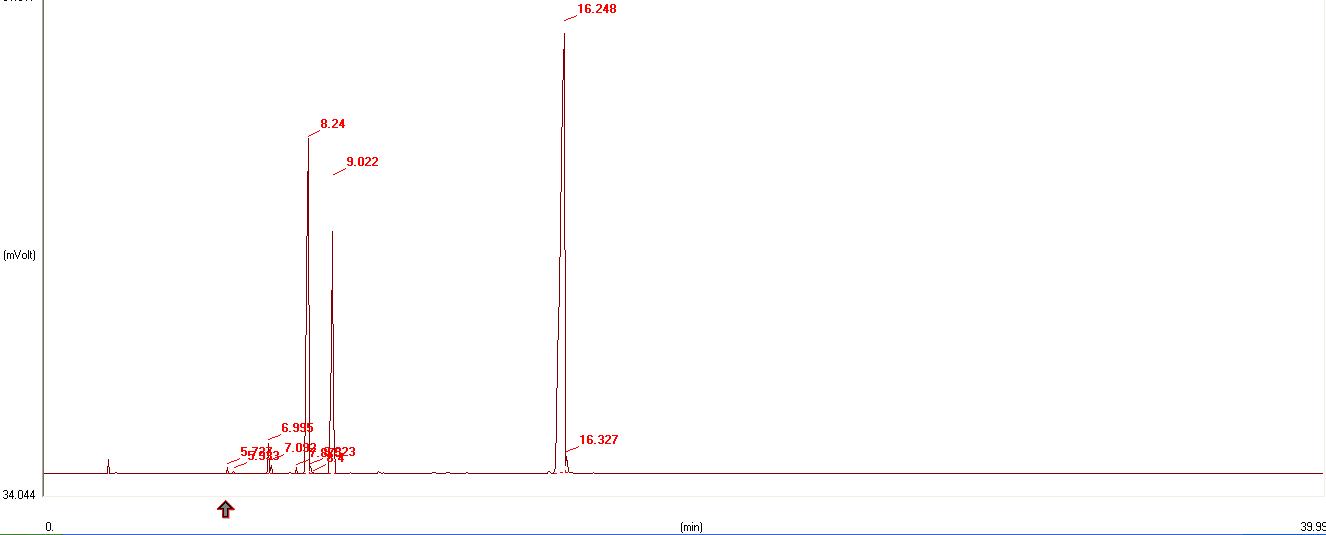

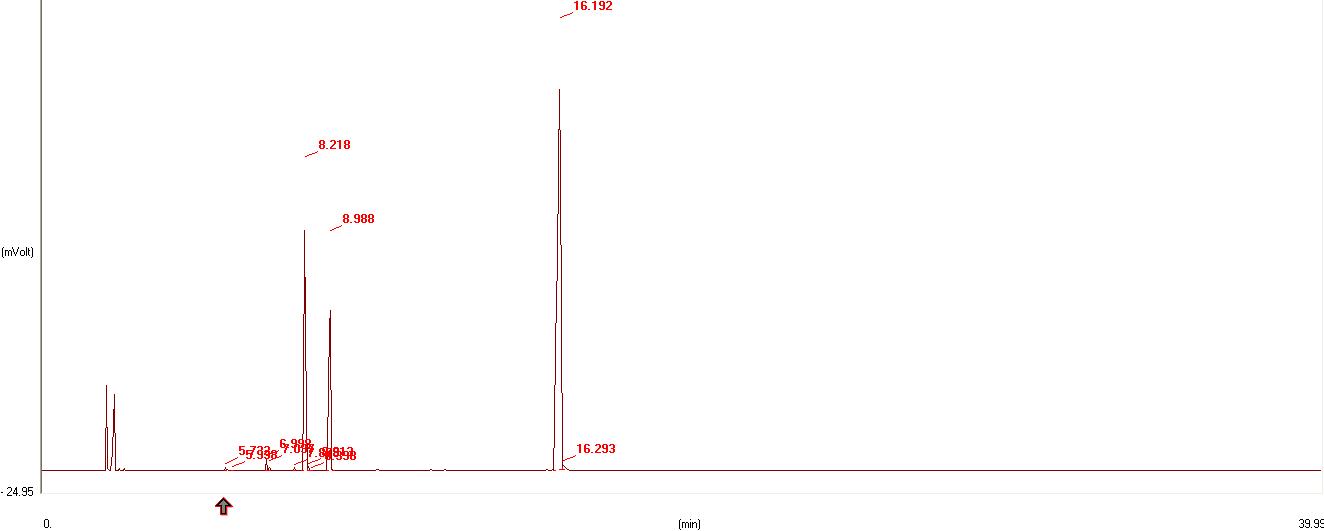


**Fig. S1.** Continued


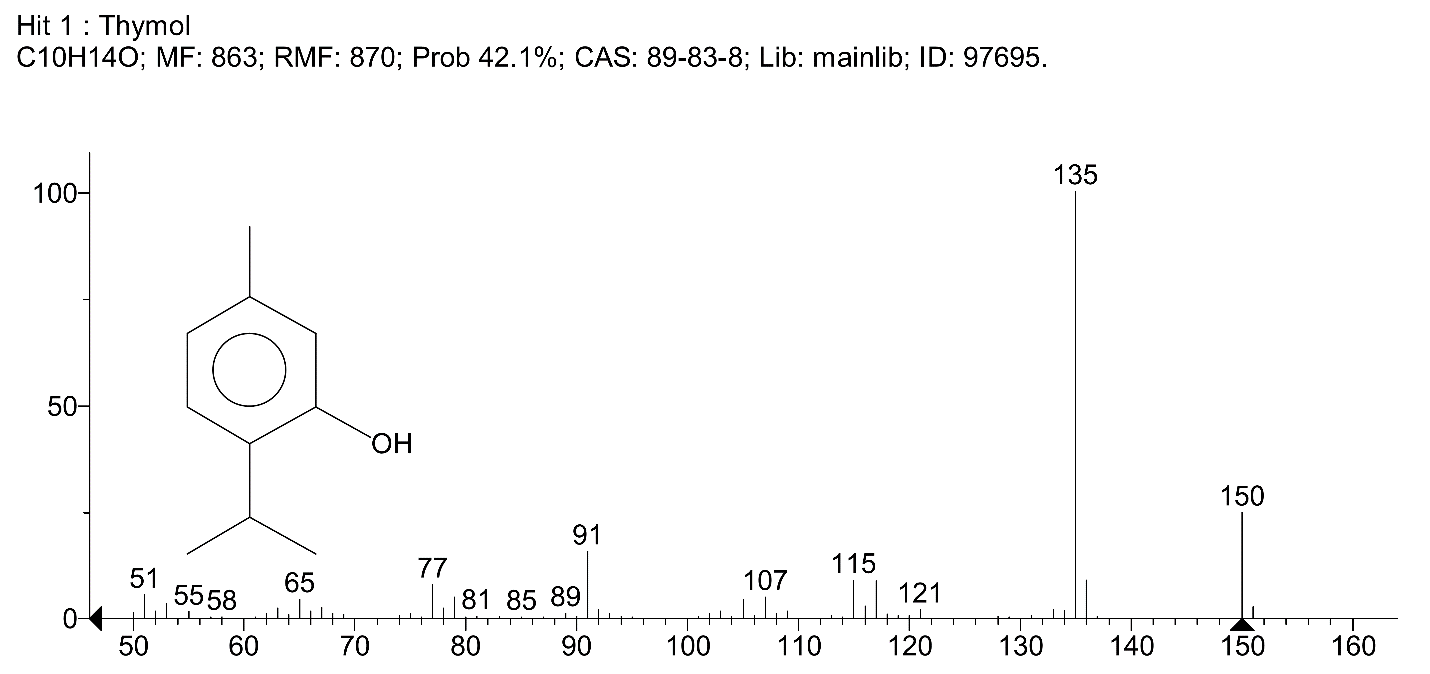


**Fig. S2.** Mass spectrum of main compound (Thymol) of 14 population of *Trachyspermum ammi.*


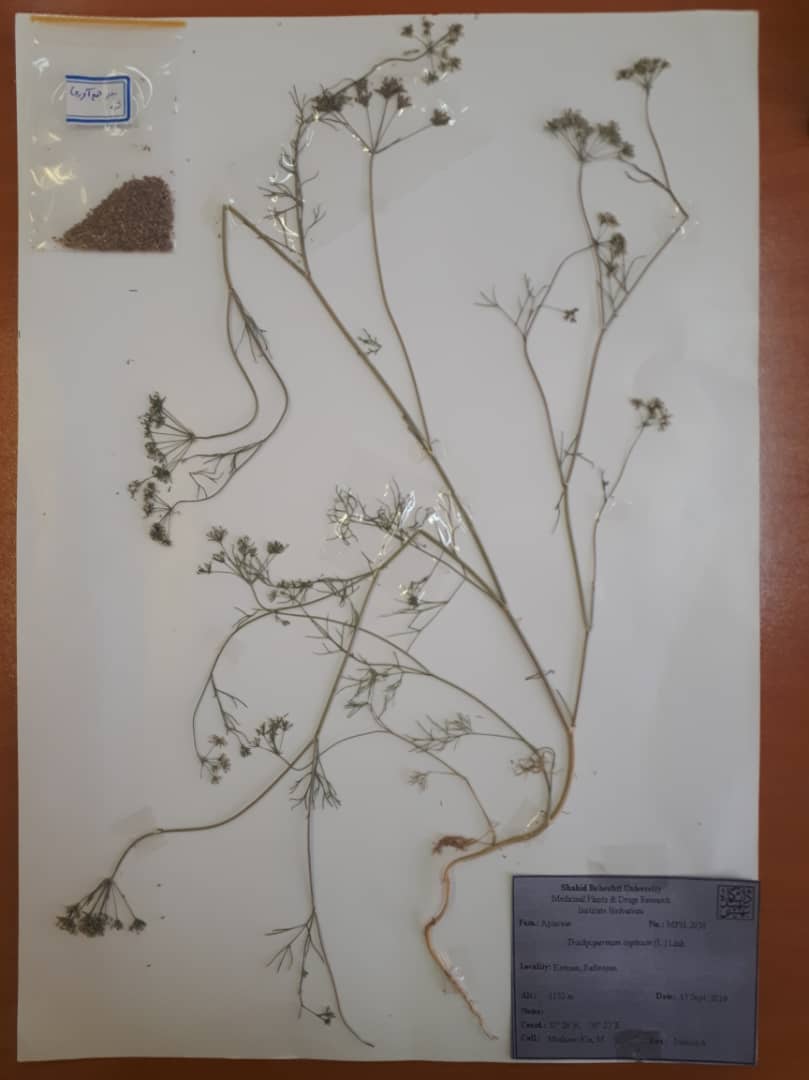


**Fig. S3.** Herbarium sample of *Trachyspermum ammi*
